# Clear zone formation in microdroplets for high-throughput screening for lactic acid bacteria

**DOI:** 10.3389/fmicb.2024.1452573

**Published:** 2024-09-18

**Authors:** Koji Mori, Mizuki Watanabe, Keiko Nanri, Satoko Matsukura, Yuri Ota, Nobuyuki Homma, Naohiro Noda

**Affiliations:** ^1^NITE Biological Resource Center (NBRC), National Institute of Technology and Evaluation (NITE), Chiba, Japan; ^2^Biomedical Research Institute, National Institute of Advanced Industrial Science and Technology (AIST), Ibaraki, Japan; ^3^On-chip Biotechnologies Co., Ltd., Tokyo, Japan

**Keywords:** water-in-oil droplet, microfluidics, clear zone, lactic acid bacteria, high-throughput screening

## Abstract

Droplet microfluidic-based technology is a powerful tool for biotechnology, and it is also expected that it will be applied to culturing and screening methods. Using this technology, a new high-throughput screening method for lactic acid bacteria was developed. In this study, the conventional culture of lactic acid bacteria that form clear zones on an agar medium was reproduced in water-in-oil droplets, and only the droplets in which lactic acid bacteria grew were collected one by one. Using this method, the specific recovery of *Lactiplantibacillus plantarum* from a mixture of *L. plantarum* and *Escherichia coli* and the acquirement of lactic acid bacteria from an environmental sample were successful. This method could be applied to various conventional screening methods using the clear zone as a microbial growth indicator. This has expanded the possibilities of applying droplet microfluidic-based technology to microbial cultivations.

## 1 Introduction

Droplet microfluidics-based technology has been recognized as a high-throughput screening method that can analyze more than 1 million samples at once (Theberge et al., 2010; Guo et al., 2012; Rosenfeld et al., 2014). The aqueous droplets are uniformly generated in oil at a rate of 1,000 per second using a microfluidic device, and the individual droplet is used as a vessel for the reaction of enzymatic and genetic analyses.

Recently, this technology has been applied to the cultivation of various microorganisms; that is, the microbial cells are encapsulated in a droplet one by one and cultured separately. The cultivations using droplet have the advantage of allowing individual cells to grow without direct interference with each other. Due to its characteristics, this has the potential to become a powerful tool for various screening methods. Some representative bacteria and yeasts, such as *Escherichia coli*, *Bacillus subtilis*, *Lactococcus lactis*, *Saccharomyces cerevisiae*, and *Phaffia rhodozyma*, have been confirmed to grow in droplets (Boitard et al., 2015; Chen et al., 2017, 2018; Hernandez-Valdes et al., 2020; Mahler et al., 2015; Kaminski et al., 2016; Zhang et al., 2017), and the cultivation in droplets is at a stage where it can be applied to various applications. Yin et al. (2022) reported that by using the cultivation in droplets, the diversity and evenness of gut bacteria obtained from animal feces was significantly increased compared to a traditional cultivation method. Isozaki et al. (2020) demonstrated the sorting of *S. cerevisiae* in droplets based on their growth rate differences by a combination of a sequentially addressable dielectrophoretic array sorter. In filamentous fungi, the cultivation of *Trichoderma reesei* in droplets also facilitated genetic manipulation and was revealed to have the potential as a powerful tool for strain modification (Luu et al., 2022).

While the culturability of various microorganisms in droplets has been demonstrated, there are still few examples of its application. One of the reasons for this is the difficulty of the technology in detecting the desired droplets among a million droplets. In addition, since it is a high-throughput method, its detection is meaningless unless it is fast. Ota et al. (2019) developed the detection method of droplets containing growing bacteria using a fluorescent nucleic acid probe. In this method, droplets are mixed with fluorescence resonance energy transfer (FRET)-based RNA probes in advance. The RNase that accompanies bacterial growth cleaves the probes, thereby identifying only droplets in which bacteria have grown. By using this method, Saito et al. (2021) successfully obtained some bacteria presented in small amounts in the sample. Nakamura et al. (2022) developed a peptidase substrate consisting of a dipeptide and 7-aminocoumarin-4-acetic acid, and successfully detected the droplets in which bacteria with dipeptidyl peptidase activity grew Two cases have been reported regarding lactic acid bacteria: Chen et al. (2017) obtained *L. lactis* mutants which produce riboflavin efficiently from the mutagenesis library; Hernandez-Valdes et al. (2020) isolated *L. lactis* mutants which produce amino acids highly by using co-cultures of the growth-based sensor strain in droplets. As these examples have shown, the use of fluorescence reactions based on microbial activity can be said to be an effective method for the high-throughput detection of specific droplets. However, it is not easy to construct the detection method because it is necessary to consider the hydrophilicity/hydrophobicity, toxicity, fading property, timing of addition, etc. of the fluorogenic compound.

**Figure 1:**
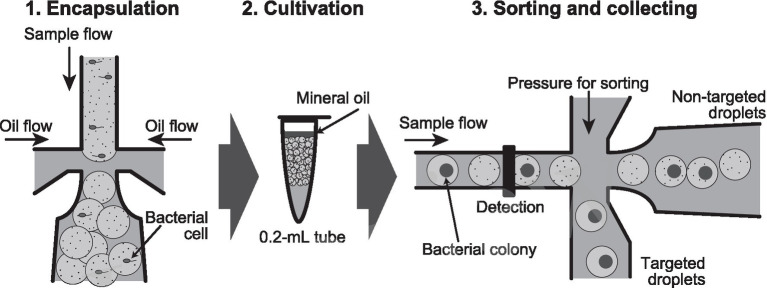
Flow chart of the high-throughput screening method of lactic acid bacteria from environment samples by using water-in-oil droplets. (1) Encapsulation of bacterial cell into 100 μm-diameter (0.5 nL volume) water-in-oil droplets. (2) Cultivation of the droplets in a 0.2 mL tube for bacterial colony formation. (3) Sorting of the droplets in which CaCO_3_ precipitate disappears, and then collecting of the targeted droplets one by one into a 96-well microplate.

Lactic acid bacteria are a group of bacteria whose industrial usefulness is associated with a variety of metabolites and bacterial cell components at the genus, species, and strain levels. The media containing CaCO_3_ have been used for their isolation, and their colonies have been detected using the clear zones associated with acid production as an indicator (e.g., Stephen and Saleh, 2023). Although this cultivation method detects anaerobic acid-producing bacteria as well as lactic acid bacteria, a lot of lactic acid bacteria have been isolated so far. In this study, we reproduced this conventional cultivation of lactic acid bacteria in water-in-oil droplets, and then only the droplets in which lactic acid bacteria grew were obtained in high-throughput with a sorter. As shown in Figure 1, our goal was to encapsulate one cell in a droplet, and after cultivation, to obtain only droplets in which CaCO_3_ had disappeared due to lactic acid bacterium colony formation, one by one, in a high-throughput manner.

## 2 Methods

### 2.1 Bacterial strains and growth conditions

*Escherichia coli* NBRC 3301 (strain K-12) and *Lactiplantibacillus plantarum* NBRC 3070 were used as model bacteria. For the pre-cultivation of *E. coli*, LB medium containing 10 g L^−1^ Hipolypepton (Fujifilm Wako, Osaka, Japan), 5 g L^−1^ yeast extract, and 10 g L^−1^ NaCl was used. For the other cultivation, GYP medium was used, and the medium contained: 10 g L^−1^ glucose, 10 g L^−1^ yeast extract, 5 g L^−1^ Hipolypepton, 2 g L^−1^ beef extract, 2 g L^−1^ sodium acetate, 0.2 mg L^−1^ MgSO_4_·7H_2_O, 0.01 mg L^−1^ MnSO_4_·5H_2_O, 0.01 mg L^−1^ FeSO_4_·7H_2_O, 0.01 mg L^−1^ NaCl, and 0.5 g L^−1^ Tween 80. Pre-cultivation of *E. coli* and *L. plantarum* were performed in liquid media. For their droplet cultivations, CaCO_3_ and agar were added in media. When CaCO_3_ and agar were used for the droplet cultivations, 0.25%(w/v) CaCO_3_ (Hayashi Pure Chemical, Osaka, Japan) and 1.5%(w/v) NuSieve^®^ GTG^®^ Agarose (Lonza, Basel, Switzerland) were added as the final concentrations. All cultivations were conducted under static conditions at 30°C. For anaerobic cultivations, the AnaeroPack system (Mitsubishi Gas Chemical, Tokyo, Japan) was used.

### 2.2 Preparation of an environmental sample

*Sphyraena pinguis* was obtained at Tokyo Bay in November 2022. The contents of approximately 1 gram of an intestinal tract were taken out with a spatula, homogenized, suspended in 0.85% (w/v) NaCl solution, and then filtered through several mesh pieces (pore sizes 2000, 710, 250, 100, and 53 μm; Nonaka Rikaki, Tokyo, Japan) to remove large solid particles. Twenty millilitres of the final solution containing 0.85%(w/v) NaCl and 10%(w/v) glycerol was divided into 1 mL portions and stored at −80°C until use. This portion was pelleted by centrifugation and resuspended in the droplet medium for use.

### 2.3 Microscopy and cell count

Water-in-oil droplets were routinely observed on slide glass with a hole by using a microscope (model AX-70; Olympus). A direct cell count was performed under a fluorescent microscope by 4′,6-diaminodino-2-phenylindole (DAPI) staining on a polycarbonate membrane filter (K020N025A; Advantec, Tokyo, Japan).

### 2.4 Droplet generation

Water-in-oil droplets were generated by using a DG8 cartridge (BioRad, CA, United States) with a On-chip^®^ Droplet Generator with a Droplet Generator temperature control unit and On-chip Droplet Generator soft ver.1.2.0 (On-chip Biotechnologies, Tokyo, Japan). For the oil phase, 008-FluoroSurfactant-5wtH-10 mL (On-chip Biotechnologies) consisting of Novec 7500 Engineered Fluid fluorinated oil (NT Science, Nagoya, Japan) and 5%(w/v) 008-FluoroSurfactant was used. Droplet generation conditions were a sample pressure of 30 kPa, oil pressure of 30 kPa, and temperature of 42°C, and droplets with a diameter of approximately 100 μm (equivalent to 0.524 nL volume) were generated. The number of bacteria encapsulated in a droplet theoretically follows a Poisson distribution (Collins et al., 2015). When the droplet volume is 0.524 nL, if the cell concentration is 1.91 × 10^6^ cells mL^−1^, then one cell is encapsulated in each droplet on average. Generated droplets were collected in 0.2 mL tubes and immediately chilled on ice for 20 min. After cooling, a mineral oil (Sigma-Aldrich, MO, United States) was put on the water-in-oil droplets, and then the tubes were incubated under anaerobic conditions.

### 2.5 Droplet sorting, dispensing, and scaled-up culturing

On-chip^®^ Droplet Selector (On-chip Biotechnologies) was used for droplet sorting. On-chip^®^ Droplet Selector uses a small disposable microfluidic chip as its core technology. Unlike conventional cell sorters, which a specific sheath fluid must be used, it allows for the use of any sheath fluid appropriate to the sample. Therefore, water-in-oil droplets or gel droplets can be flowed as samples. It is also possible to dispense droplets within the target range of forward scatter (FSC) or side scatter (SSC) value.

After the droplet cultivations, the oil of the water-in-oil droplets was replaced by On-chip^®^ T-Buffer (On-chip Biotechnologies) as a phosphate-buffered-saline-based buffer for the droplet sorting. The oil of the water-in-oil droplets was removed as much as possible, an equal volume of 10%(v/v) 1H,1H,2H2H-Perfluoro-1-octnol (Fujifilum Wako) in Novec 7500 Engineered Fluid fluorinated oil was added, and then the solution was mixed thoroughly. An equal volume of On-chip^®^ T-Buffer was then gently added, and after 10 min, the upper On-chip^®^ T-Buffer containing gel droplets was transferred to a new tube.

The FSC and SSC intensities of the gel droplets in the On-chip^®^ T-Buffer were analyzed, and based on their scatter plots, the desired droplets were sorted and collected one by one into a 96-well plate with an On-chip^®^ Droplet Selector and OnChipFlow software (ver.1.10). Gel droplets were suspended in On-chip^®^ T-Buffer containing 40%(v/v) Opti-prep^™^ (iodixanol; Serumwerk, Bernbkurg, Germany), and loaded into the sample reservoir of microfluidic chip (2D Chip-SD1000-w150; On-chip Biotechnologies). On-chip^®^ T-Buffer was used as a sheath fluid for sorting. Sorting conditions were followings: main push1, 3 kPa; main push2, 2.3 kPa; main pull, −0.6 kPa. The sample flow rate was less than 20 events per second. Gates on the scatter plots were arbitrarily defined to collect a droplet population of low SSC values. The gel droplet within the gated area was pushed out into the collection reservoir of the microfluidic chip and dispensed one by one into a 96-well plate.

After the collection of individual gel droplets into a 96-well plate, a 200 μL of GYP medium was added, and the plate was anaerobically incubated at 30°C for 2 days. After confirming the growth turbidity, a 2 μL culture was dropped in the GYP medium plate supplemented with CaCO_3_ and the plate was anaerobically incubated at 30°C for 1 day to allow colony formation. Microbial colonies with clear zones were scraped with a loop, suspended in 75%(v/v) ethanol and GYP medium containing 10%(w/v) glycerol, and stored at −80°C until analysis for MALDI-TOF MS and 16S rRNA gene sequence, respectively.

### 2.6 MALDI-TOF MS analysis and 16S rRNA gene sequencing

Confirmation that it was lactic acid bacteria was first done by forming a clear zone at the above scaled-up culture. All the lactic acid bacterium cultures were grouped by the analysis of matrix-assisted laser desorption ionization-time-of-flight mass spectrometry (MALDI-TOF MS), and then the 16S rRNA gene sequences of representative strains were determined.

For the MALDI-TOF MS analysis, a pelleted cell was obtained from the above colony suspension by centrifugation. Twenty microlitres of 70%(w/v) formic acid was added to the pellet, and after a mixing, an equal volume of acetonitrile was added. After centrifugation at 12,000 rpm for 5 min, the supernatant was transferred to a new tube. One μL of supernatant was applied to a target plate (MSP BigAnchor 96 BC; Bruker, Bremen, Germany) and dried. Then, 1 μL of MALDI matrix (HCCA; Bruker) was applied to each sample and dried. Measurements were performed with a Microflex^™^ LT (Bruker). Mass spectra were compared using MALDI Biotyper Ver.3.1 software and the dendrogram constructed based on the spectra using the BioTyper MSP Dendrogram Creation Standard Method.

For the 16S rRNA gene sequence determination, genomic DNA was prepared by using PrepMan^™^ Ultra Sample Preparation Reagent (Thermo Fisher Scientific, MA, United States), and PCR amplification was performed by the following conditions: forward primer 9F (5′-GAGTTTGATCCTGGCTCAG-3′) and reverse primer 1510R (5′-GGCTACCTTGTTACGA-3′); PCR reaction (10 μL) contained 0.2 unit KOD FX DNA polymerase, 5 μL buffer, 0.2 mM dNTP (Toyobo, Osaka, Japan), 0.3 μM forward and reverse primers, 1 μL sample. Thermal cycling conditions consisted of pre-heating at 94°C for 1 min, followed by 30 cycles of 98°C for 10 s, 55°C for 30 s, and 68°C for 1.5 min, and a final 1 min extension step at 72°C for 1 min. PCR amplicon was purified using AMPure XP (Beckman Coulter, CA, United States) and sequenced using the BigDye Terminator ver.3.1 Cycle Sequencing Kit (Thermo Fisher Scientific) under previously described conditions (Mori et al., 2008) with a 3500 genetic analyzer (Thermo Fisher Scientific). To identify the obtained sequences, BLAST analysis was performed using the NCBI database.^1^

## 3 Results and discussion

### 3.1 Micro-colony formation in a water-in-oil droplet

**Figure 2:**
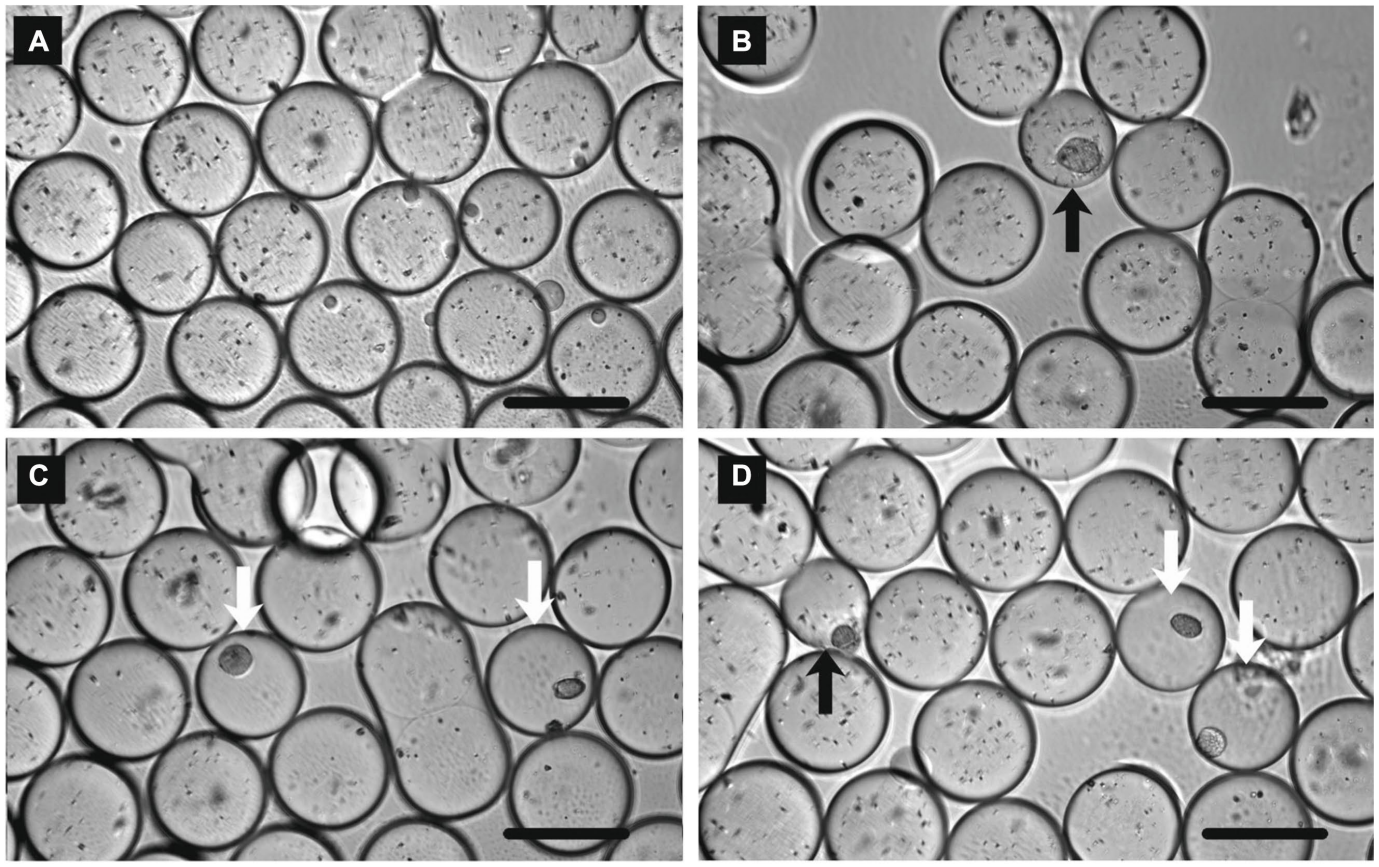
Micrographs of water-in-oil droplets after 6 days cultivation with **(A)** non-inoculum, **(B)** *Escherichia coli*, **(C)** *Lactiplantibacillus plantarum*, and **(D)** *L. plantarum* and *E. coli*. Open and solid arrows indicate the bacterial colonies of *L. plantarum* and *E. coli*, respectively. Scale bar = 100 μm.

To demonstrate colony formation in a water-in-oil droplet, *L. plantarum* and *E. coli* were used as model bacteria. *L. plantarum* is a facultatively anaerobic lactic acid bacterium, and the clear zone around the colony could be observed on the agar plate of GYP medium containing CaCO_3_ after the 1-day anaerobic cultivation (data not shown). In the case of *E. coli*, the clear zone was not confirmed under the same conditions (data not shown). To encapsulate one cell in a 0.5 nL water-in-oil droplet, pre-cultures of *E. coli* and *L. plantarum* were diluted with fresh media to 2.62 × 10^5^ cells mL^−1^ and 3.43 × 10^5^ cells mL^−1^, respectively, and then the droplets were prepared and cultivated. After a 6-day incubation, micro-colonies of *E. coli* and *L. plantarum* (Figures 2B,C) were observed in some droplets under a microscope. Inhomogenous fine particles (CaCO_3_) were observed in the most droplets. The particles of CaCO_3_ disappeared only in the droplets in which the colonies of *L. plantarum* appeared (open arrows in Figure 2C), but remained in the droplets in the case of non-inoculum (Figure 2A) and *E. coli* cultivation (solid arrow in Figure 2B). When the mixture of pre-cultures of *L. plantarum* and *E. coli* was encapsuled in the droplets, the colonies with both the disappeared and remaining particles of CaCO_3_ (open and solid arrows, respectively, in Figure 2D) could be observed, suggesting that the particles disappeared only in the droplets in which the colonies of *L. plantarum* formed. From the above, we succeeded in reproducing the detection of lactic acid bacteria by clear zone formation in water-in-oil droplets.

Under the above conditions, although the 18% of droplets contained cells theoretically, *L. plantarum* colonies were observed in the 3–5% droplets under a microscope, and this ratio was less than one-third of the theoretical value for the encapsulating cells. A possible reason for this was that some cells could not form a colony from a single cell. When the number of encapsulated *L. plantarum* cells was increased 10-fold, many droplets in which the CaCO_3_ particles disappeared but no colonies appeared were also observed under a microscope (data not shown). Since it has been reported that substances move between droplets by diffusion, and the movement is particularly large when the droplets are dense (Courtois et al., 2009; Gruner et al., 2016), it was inferred that the *L. plantarum-*colonized droplets influenced the empty droplets. This indicates that the dilution ratio is an important factor in detecting the droplets in which lactic acid bacteria grow.

Although a water-in-oil droplet is generally considered to be stable for a long time (Gruner et al., 2015), the droplets which we prepared often aggregated and stuck together tightly after incubation. This phenomenon was suppressed to some extent by adding mineral oil on the water-in-oil droplets, so it was thought that dryness was one of the causes. On the other hand, it may be that the tween 80 in the GYP medium reacted with the surfactant, 008-FluoroSurgactant, and affected the stability of droplets (Jiao and Burgess, 2003). Surfactants such as tween 80 are commonly added to lactic acid bacterium culture media to protect the cells, improve its nutrient uptake, and promote its growth (Aljaloud et al., 2019). The type and concentration of surfactant used for the water-in-oil droplets of lactic acid bacteria are one of future issues to investigate.

### 3.2 Specific detection and separation with a sorter of gel droplets in which lactic acid bacteria grew

Initially, the fluorinated oil with 5%(w/v) 008-FluoroSurfactant was used as a sheath fluid for sorting the droplets, as well as for the culture, but the microfluidic channel of the sorter was often clogged with the aggregated droplets. Therefore, On-chip^®^ T-Buffer as a phosphate-buffered-saline-based buffer was used as a sheath fluid to successfully sort, detect, and collect the droplets. In this study, water-in-oil droplets were used until the cultivation, and after that, gel droplets were used by the replacement of buffer. Unfortunately, however, the need to replace the sheath fluid took away one of the benefits of water-in-oil droplets: even if the droplets are negative in the first sort, microorganisms such as slow-growers can be recovered by re-cultivating and sorting repeatedly (Saito et al., 2021).

**Figure 3:**
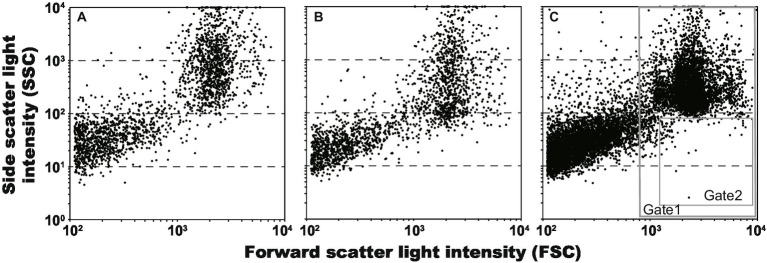
Scatter plots of gel droplets after 7 days cultivations, and gate setting for obtaining of lactic acid bacterium cultures. The Gates 1 and 2 were defined for removing anything other than microdroplets and for obtaining microdroplets of lactic acid bacteria, respectively. **(A)** *Escherichia coli*, 2,125 events in 29 s (73.3 event s^−1^); **(B)** *Lactiplantibacillus plantarum*, 1,971 events in 25 s (78.8 event s^−1^); and **(C)** *L. plantarum* and *E. coli*, 10,000 events in 608 s (16.5 event s^−1^).

Regarding when On-chip^®^ T-Buffer was used as the sheath fluid, scatter plots of gel droplets after 7-day cultivations of *E. coli* (2,125 events in 29 s; 73.3 event s^−1^) and *L. plantarum* (1,971 events in 25 s; 78.8 event s^−1^) are shown in Figures 3A,B, respectively. The plots were divided into 2 main groups, and the group with high FSC values—accounting for half of the total—was the droplet group. The group with low FSC values was that for mineral oil and the remains of broken gel droplets. The SSC values, indicating the complexity inside, of some *L. plantarum* droplets decreased compared to that of the *E. coli* droplets. This suggested that the SSC values decreased due to the disappearance of the CaCO_3_ particles associated with *L. plantarum* colony formation. Therefore, we tested whether it was possible to collect only the droplets in which *L. plantarum* grew using a sorter from the 7-day-incubated droplets prepared using the mixture of *E. coli* and *L. plantarum*. The scatter plot of the droplets of the mixture (10,000 events in 608 s; 16.5 event s^−1^) is shown in Figure 3C. Because the 1.5–2.5% droplets were suggested by the microscopic observation of previous test to indicate *L. plantarum* growth, only droplet events were selected from all events (Gate 1 in Figure 3C), and 1% of droplets with low SSC values in all droplets (Gate 2 in Figure 3C) were collected in a 96-well plate. Consequently, 96 droplets were collected into the 96-well plate from the 33,577 events in 2,122 s (15.8 event s^−1^). Through the scale-up cultivation and clear zone formation, it was confirmed that *L. plantarum* grew in 95 out of 96 droplets. The above results indicated that the droplets containing one microbial cell could be reliably fractionated by the oil, and that only droplets in which the CaCO_3_ particles had disappeared could be selectively collected using the sorter.

### 3.3 Isolation of lactic acid bacteria from an environmental sample

**Figure 4:**
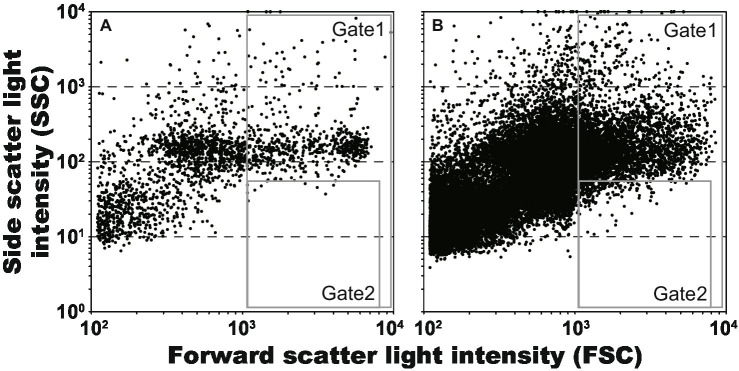
Scatter plots of gel droplets after 7 days cultivations, and gate setting for obtaining of lactic acid bacterium cultures. The Gates 1 and 2 were defined for removing anything other than microdroplets and for obtaining microdroplets of lactic acid bacteria, respectively. **(A)** *Lactiplantibacillus plantarum*, 5,130 events in 61 s (84.1 event s^−1^); and **(B)** gut of *Sphyraena pinguis*, 26,657 events in 906 s (29.4 event s^−1^).

We attempted to obtain lactic acid bacteria from an environmental sample using the procedures developed in this study. The content of the intestinal tract of *S. pinguis* was used as an environmental sample, and approximately 0.05 g mL^−1^ of this was encapsulated in the droplets. Because the proportion of lactic acid bacteria contained in the sample was unknown, the collecting area in the scatter plot was firstly determined using the model bacterium, *L. plantarum*. As shown in Figure 4A, by using the scatter plot of *L. plantarum* droplets (2,125 events), the droplet area (Gate 1) and then the collecting area as 1% of droplets with low SSC values (Gate 2) were determined. Subsequently, the gel droplets of the 7-day cultivation of the content of the intestinal tract were sorted and the droplets in Gate 2 were collected (Figure 4B). Consequently, 96 droplets were collected from the 26,657 events in 906 s (29.4 event s^−1^), and all the collected droplets were confirmed to be lactic acid bacteria through the scale-up cultivation and clear zone formation. Although the obtained lactic acid bacteria were divided into several groups by the MALDI-TOF MS analysis, all of them had 100% 16S rRNA gene sequence identity with the type strain of *Leuconostoc citreum* (LC096222). The genus *Leuconostoc* has been isolated mainly from food and humans, and their habitat are the intestines of animals (Holzapfel et al., 2015).

By using the procedures developed in this study (Figure 1), we could obtain lactic acid bacteria from an environmental sample. The FSC values in Figure 4 were more spread out horizontally than the results in Figure 3. It is currently unclear whether this reason is due to size fluctuations during the droplet generation or fluctuations in detected values during the sorting. By spreading horizontally, Gates 1 and 2 in Figure 4 were set to ensure that non-droplets were avoided. As a result, because only a limited range of droplets could be collected, all of the collected droplets were lactic acid bacteria but the diversity may have been low. More efforts are needed to increase the diversity of lactic acid bacteria.

## 4 Conclusion

In this study, we were able to reproduce the conventional cultivation method of lactic acid bacteria in water-in-oil droplets, and in high-throughput collect only the droplets in which they grew. In this procedure, the dilution rate of the environmental sample is one of important factors. The type and concentration of surfactant used for their cultivation and droplet generation is a matter for future consideration. Also, in order to collect a variety of lactic acid bacteria from varying environments, it is necessary to refine the details of the conditions such as gating of droplet selection.

This high-throughput method of collection of lactic acid bacteria from varying environments can be detected by a simple decrease in turbidity, so it is thought to be applicable to conventional isolations using clear zones. Applying droplet microfluidics-based technology to cultivation methods is expected to expand the possibilities of microbial screening.

## Data Availability

The original contributions presented in the study are included in the article, further inquiries can be directed to the corresponding author.

## Footnotes

^1^ www.ncbi.nlm.nih.gov/BLAST/
